# Effect of Long-Distance Domestic Travel Ban Policies in Japan on COVID-19 Outbreak Dynamics During Dominance of the Ancestral Strain: Ex Post Facto Retrospective Observation Study

**DOI:** 10.2196/44931

**Published:** 2024-04-22

**Authors:** Junko Kurita, Yoshitaro Iwasaki

**Affiliations:** 1 Department of Nursing Faculty of Sports & Health Science Daitobunka University Higashimatsuyama-shi Japan; 2 Iwasaki Industrial Corporation Kagoshima Japan

**Keywords:** airport users, COVID-19, effective reproduction number, Go To Travel campaign, hotel visitors, mobility, long-distance travel, infection control, lockdown, travelling, travel, pandemic

## Abstract

**Background:**

In Japan, long-distance domestic travel was banned while the ancestral SARS-CoV-2 strain was dominant under the first declared state of emergency from March 2020 until the end of May 2020. Subsequently, the “Go To Travel” campaign travel subsidy policy was activated, allowing long-distance domestic travel, until the second state of emergency as of January 7, 2021. The effects of this long-distance domestic travel ban on SARS-CoV-2 infectivity have not been adequately evaluated.

**Objective:**

We evaluated the effects of the long-distance domestic travel ban in Japan on SARS-CoV-2 infectivity, considering climate conditions, mobility, and countermeasures such as the “Go To Travel” campaign and emergency status.

**Methods:**

We calculated the effective reproduction number R(*t*), representing infectivity, using the epidemic curve in Kagoshima prefecture based on the empirical distribution of the incubation period and procedurally delayed reporting from an earlier study. Kagoshima prefecture, in southern Japan, has several resorts, with an airport commonly used for transportation to Tokyo or Osaka. We regressed R(*t*) on the number of long-distance domestic travelers (based on the number of airport limousine bus users provided by the operating company), temperature, humidity, mobility, and countermeasures such as state of emergency declarations and the “Go To Travel” campaign in Kagoshima. The study period was June 20, 2020, through February 2021, before variant strains became dominant. A second state of emergency was not declared in Kagoshima prefecture but was declared in major cities such as Tokyo and Osaka.

**Results:**

Estimation results indicated a pattern of declining infectivity with reduced long-distance domestic travel volumes as measured by the number of airport limousine bus users. Moreover, infectivity was lower during the “Go To Travel” campaign and the second state of emergency. Regarding mobility, going to restaurants, shopping malls, and amusement venues was associated with increased infectivity. However, going to grocery stores and pharmacies was associated with decreased infectivity. Climate conditions showed no significant association with infectivity patterns.

**Conclusions:**

The results of this retrospective analysis suggest that the volume of long-distance domestic travel might reduce SARS-CoV-2 infectivity. Infectivity was lower during the “Go To Travel” campaign period, during which long-distance domestic travel was promoted, compared to that outside this campaign period. These findings suggest that policies banning long-distance domestic travel had little legitimacy or rationale. Long-distance domestic travel with appropriate infection control measures might not increase SARS-CoV-2 infectivity in tourist areas. Even though this analysis was performed much later than the study period, if we had performed this study focusing on the period of April or May 2021, it would likely yield the same results. These findings might be helpful for government decision-making in considering restarting a “Go To Travel” campaign in light of evidence-based policy.

## Introduction

Important features of countermeasures against the COVID-19 outbreak in Japan were restrictions such as staying at home, wearing a mask, holding virtual meetings at organizations, and conducting contact tracing. All these measures were implemented on a voluntary basis; that is, the government strongly recommended such measures but none was required as a matter for law enforcement. Therefore, lockdowns such as those that occurred in the United States or some European countries, entailing enforced laws, never occurred in Japan. Even though these countermeasures were recommended by the government without enforced laws, aside from laws implemented at border controls and for quarantines, most Japanese people cooperated with the recommendations voluntarily.

**Figure 1:**
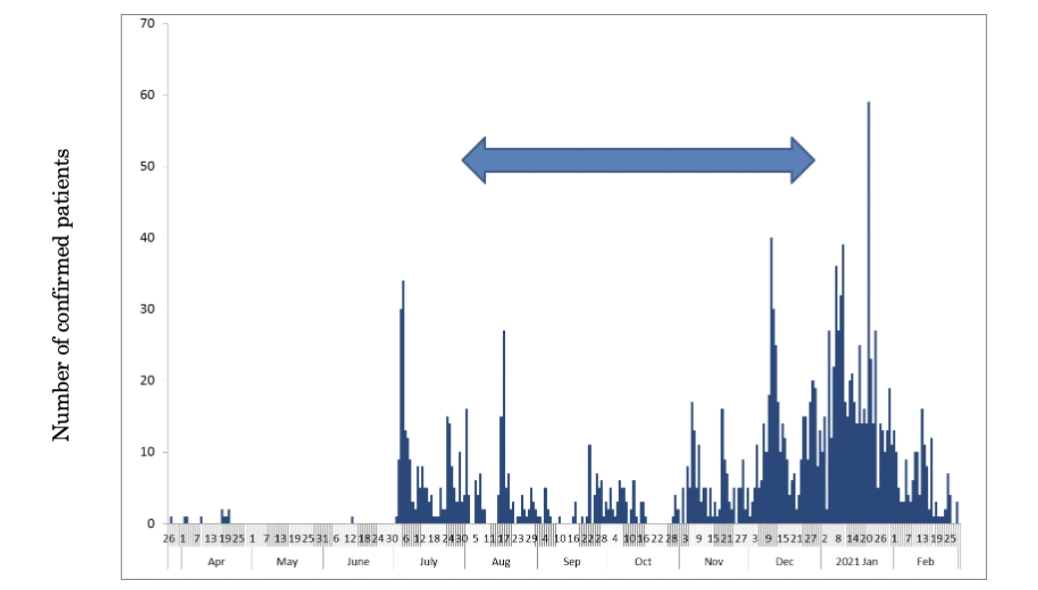
Numbers of newly confirmed COVID-19 cases in Kagoshima prefecture, Japan, from March 26, 2020, to the end of February 2021. Bars represent the epidemic curve showing the numbers of patients by onset date. The arrow indicates the period during which the "Go To Travel" campaign was in effect.

At the beginning of the COVID-19 outbreak in Japan, school closures and voluntary event cancellations were required from February 27 through March of 2020. Large commercial events were also cancelled. Subsequently, a state of emergency was declared from April 7 through May 25, with voluntary restrictions against leaving the home and requiring the shutting down of businesses serving customers. During this period, the first peak in the outbreak was reached on April 3, 2020. Another peak then emerged on July 29, as shown in Figure 1. The so-called “Go To Travel” campaign (GTTC) started on July 22, 2020, with 50% subsidized travel and coupons issued for shopping at tourist destinations. The policy was aimed at reinforcing sightseeing businesses, even though such a measure entailed the possibility of expanding the outbreak. Thereafter, the GTTC continued through December 2020, by which time a third wave of infections had emerged, which was larger than either of the prior two waves. Therefore, the GTTC was implicated as the main underlying reason for the third wave [1].

Although results were mixed, some studies suggested that the spread of COVID-19 was associated with climate conditions in China [2-4]. However, other research based on cross-sectional international comparisons in European countries found no association between climate conditions and COVID-19 outbreak surge dates [5]. In Japan, high temperatures, humidity, and sunlight exposure were associated with reduced infectivity according to analyses of cross-sectional data [6]. One review [7] indicated an association of outbreaks with temperature, whereas no association was confirmed with humidity, rainfall, or even air pollution. Another cross-sectional analysis conducted in Italy [8] showed an association of COVID-19 outbreaks with air pollution. If such an association was valid for Japan, then the GTTC might not in fact be the main contributor to the occurrence of the third wave of infections in the winter of 2020-2021.

Indeed, mobility was presumed to be the main driver of outbreak dynamics, at least for the first wave in Japan [9] as well as globally [10]. In a study conducted early in the pandemic, up to April 2020, Flaxman et al [11] demonstrated that nonpharmaceutical interventions, including lockdowns, strongly reduced transmission in at least 11 European countries. However, another study including 131 countries found that the introduction and relaxation of lockdowns or movement restrictions had only limited the effects on infectiousness, except for public event bans, although their data were limited to the end of July 2021 [12]. Another study indicated that strict movement restrictions in Argentina imposed as of March 2020 were effective at reducing mobility, but not for mitigating the outbreak [13]. These mixed results suggest that such countermeasures might not have significantly affected the infectivity of SARS-CoV-2.

By contrast, there is abundant evidence demonstrating the effects of international travel and restrictive policies on pandemic dynamics, mainly focusing on the effects of long-distance domestic travel [14-17]. However, despite a report indicating that 80% of patients with COVID-19 on an island in Canada had acquired a travel-related infection from July 1, 2020, to May 31, 2021 [18], in Japan, only 12% of patients had a recent history of international travel in the very early phase of the pandemic from January 13 to March 31, 2020 [19].

Some studies have emphasized associations among international trade and outbreak sizes [20,21]. Although the traded goods are likely not the source of infectiousness, international trade volumes might be related to outbreak size, as this volume can reflect the movements of business representatives accompanying international trade. Unfortunately, these two studies were based on cross-sectional analyses and therefore it was not possible to isolate the effects of the number of travelers from the effects of high population densities or air pollution discharged by manufacturing industries. Therefore, these studies did not provide sufficient evidence to confirm that travelers were mainly responsible for expanding the outbreak. Moreover, immediately after the pandemic was declared, most international borders had been closed in principle and almost all planned face-to-face meetings related to international trade were held as virtual meetings [22].

Countermeasures against the spread of COVID-19 differed considerably with respect to international and domestic travel, as the former involves border controls and restrictive quarantines, which might be more effective at reducing transmission [23-26]. By contrast, domestic travel recommendations might be limited to voluntary restrictions against going out and long-distance domestic travel. Even in the case of a long-distance domestic travel ban, a chain of short-distance trips to a neighboring city, including commuting to a school, workplace, or shopping center, might ultimately act to transmit viruses over long distances, given some delay. In this sense, experience and evidence related to international travel might not be directly applicable to long-distance domestic travel.

To our knowledge, no study has examined the impact of long-distance domestic travel on outbreak situations in rural areas. One might expect that such information might be less available for epidemiological analysis. Although annual or monthly data related to travelling or sightseeing might be generally available, such records are generally not widely available. Moreover, these data would be quite aggregated, and the number of data points would likely be too small to support sufficient power for statistical analyses considering short time periods of less than 1 year. Fortunately, epidemiologists, statisticians, and companies managing resort hotels and buses to airports in rural areas can provide travel-related data collaboratively. In fact, daily data of bus users from airports and visitors to these hotels are available for many areas in Japan. Therefore, the hypothesis that sightseeing visitors and long-distance domestic travelers were largely responsible for spreading the virus and contributed to COVID-19 outbreaks in rural areas can be tested directly. This hypothesis served as the rationale for ceasing the GTTC and for banning long-distance domestic travel during the first and second states of emergency in Japan. Nevertheless, this rationale has neither been analyzed nor confirmed to date.

**Figure 2:**
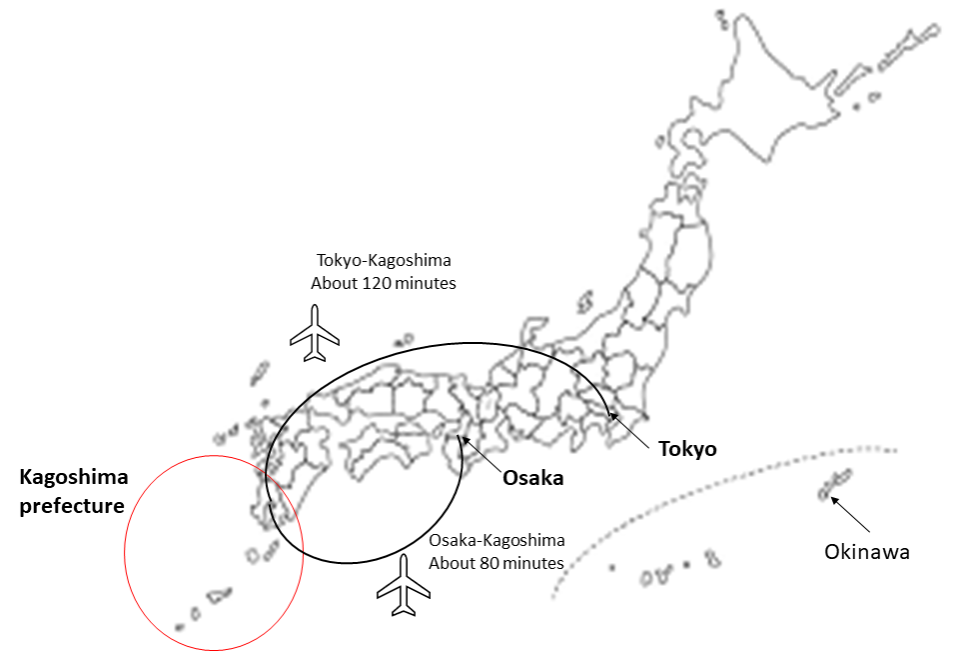
Map of Japan with Kagoshima prefecture indicated in the red circle and the airport routes to the main tourism cities for long-distance domestic travel marked.

Therefore, the objective of this study was to directly examine the hypothesis that long-distance domestic travel was responsible for expanding the COVID-19 outbreak, supporting the rationale and legitimacy of the policy followed in Kagoshima prefecture, Japan. This area was selected given that Kagoshima, located north of Okinawa but in southern Japan (Figure 2), has one airport that is used for commuting to more urban areas of the country, such as Tokyo and Osaka. Moreover, collaborative data obtained from epidemiologists and from leading tourist industry companies were available for Kagoshima, offering a valuable resource for this analysis that can contribute to more insightful consideration and policy evaluation.

## Methods

### Sample and Data

Data reflecting the daily numbers of Kagoshima airport limousine bus users were provided by Iwasaki Industrial Corp, Ltd, of Kagoshima. However, the information does not completely reflect the traffic to and from the airport, as some airport users commuted to or from the airport by taxi, private car, or rental car. Moreover, some tourists visited Kagoshima without using an airline, such as by train, car, bus, or ship. Nevertheless, most tourists from Tokyo, Osaka, or other urban areas typically use airlines to visit Kagoshima. Therefore, although the extent of domestic travel could not be verified completely, we infer that the available information accurately reflects the general picture of movement during this time.

The study period was defined as June 20, 2020, through February 2021. Before this period, COVID-19 cases had been confirmed only sporadically. Therefore, the effective reproduction number R(*t*) could not be stably estimated. However, after this period, the Alpha variant strain emerged and dominated up to 35% of all cases throughout Japan by the end of March 2021 [27]. The infectiousness of the Alpha variant was estimated to be 35%-90% higher than that of the ancestral strain [28-31]. Such a large difference in virus characteristics could affect the estimations related to our study objectives. Therefore, we limited the study period to the time prior to the emergence of the Alpha variant strain.

### Variables

Climate variables considered were average temperature (measured in degrees Celsius) and relative humidity data for Kagoshima during the day; these data were obtained from the Japan Meteorological Agency [32].

We also used mobility data provided by Google, which includes data for six types of locations: restaurants, shopping malls or amusement centers, grocery stores or pharmacies, parks, transition areas, workplaces, and homes [33]. These data show mobility comparisons according to a base day; a value of 100 was assigned if the number of people recorded for a given type of place was the same as that recorded on the base day.

Additionally, we considered the impact of major countermeasures against the pandemic implemented in Japan: two emergency state declarations, the GTTC, and school closure and voluntary event cancellation. The latter measure extended from February 27 through March in 2020, requiring school closure and cancellation of voluntary events, along with cancellation of private meetings. The first state of emergency was declared on April 7, 2020, which ceased at the end of May. This involved required school closures, shutting down of some businesses, and voluntary restrictions against going out. For Kagoshima prefecture, the state of emergency spanned from April 16 to May 14, 2020. To subsidize travel and shopping at tourist destinations, the GTTC started on July 22, 2020, and was halted at the end of December 2020.

The second state of emergency was declared on January 7, 2021, and continued until March 21, 2021, for the 11 most-affected prefectures in Japan. This countermeasure required restaurant closure at 8 PM, along with voluntary restrictions against going out, but it did not require school closure. During the study period, the GTTC and the second state of emergency were in effect. Although this second state of emergency was not declared for Kagoshima prefecture, it was implemented in major cities, including Tokyo and Osaka.

### Models and Data Analysis

The numbers of newly confirmed COVID-19 cases each day were reported by the Kagoshima Prefecture Office from May 13, 2020, through February 2021 [34]. The effective reproduction number R(*t*) was estimated according to a previous study [35]. We first estimated the onset date of patients for whom onset dates were not reported. Letting *f*(*k*) represent the empirical distribution of the incubation period and letting *N_t_* denote the number of patients for whom onset dates were not available as published at date *t*, the number of patients for whom the onset date was known is designated *t*-1. The number of patients with onset date *t*-1 for whom onset dates were not available was estimated as *f*(1)*N_t_*. Similarly, patients with onset date *t*-2 and for whom onset dates were not available were estimated as *f*(2)*N_t_*. Therefore, the total number of patients for whom the onset date was not available, given an onset date of *s*, was estimated as Σ_*k*=1_*f*(*k*)*N_s_*+*k* for the long duration extending from *s*.

Moreover, the reporting delay for published data from the Ministry of Health, Labour and Welfare of Japan might be considerable. In other words, if *s*+*k* is larger than that in the current period *t*, then *s*+*k* represents the future for period *t*. Consequently, *N_s_*_+*k*_ is not observable. Such a reporting delay engenders underestimation of the number of patients. Therefore, the formula must be adjusted to Σ_*k*=*1*_^*t*–*s*^*f(k*)*N_s_+k* /Σ_*k*=*1*_^*t*–*s*^*f*(*k*). Similarly, patients for whom the onset dates were available are expected to be affected by the reporting delay. Therefore, the formula *M_s_*|_*t*_ /Σ_*k*=*1*_^*t*–*s*^*f*(*k*) was used, where *M_s_*|*_t_* represents the reported number of patients for whom the onset date was period *s* as of the current period *t*.

We defined R(*t*) as the number of infected patients on day *t* divided by the number of patients who were presumed to be infectious. The number of infected patients was calculated from the epidemic curve by the onset date using an empirical distribution of the incubation period, which is Σ_*k*=*1*_*f*(*k*)*E_t+k_*, where *E_t_* denotes the number of patients for whom the onset date was period *t*. The distribution of infectiousness in symptomatic and asymptomatic cases *g*(*k*) was assumed to be 30% on the onset day, 20% on the following day, and 10% for the subsequent 5 days [36]. Therefore, the number of infectious patients was calculated as Σ_*k*=*1*_*g*(*k*)*E_t_*_–*k*_ and R(*t*) was defined as Σ_*k*=*1*_*f*(*k*)*E_t+k_*/Σ_*k*=*1*_*g*(*k*)*E_t_*_–*k*_*.* The empirical distributions of *f* and *g* based on actual data in Japan were obtained from an earlier report [35].

The bootstrapping procedure was applied to calculate the 95% CIs of R(*t*). We used fully replicated bootstrapping for a constant number of cases in this study period. There were *L* patients in the actual data of this study period, with numbering of the patients from the initial case to the last case. Initially, no patient was on the bootstrapped epidemic curve. If a random variable drawn from a uniform distribution of (0,1) was included in the interval [*i*/(*L*–1), (*i+*1)/(*L*–1)], then we added 1 to the onset date of the *i+*1th patient to the bootstrapped epidemic curve. We replicated this procedure *L*–1 times. Thereby, we obtained the bootstrapped epidemic curve with *L*–1 patients. We calculated R(*t*) based on the bootstrapped epidemic curve. We denote R(*t*) based on the *j*th bootstrapped epidemic curve as R(*t*)*^j^*. We repeated these processes 10,000 times to obtain R(*t*)*^j^* (*j*=1–10,000). We reordered superscripts in each *t* from min_j_{R(*t*)*^j^*} to max_j_{R(*t*)*^j^*} and denoted the reordered R(*t*)*^j^* as R(*t*)*^k^ (k=1–10,000). The estimated R(*t*) was then taken as the median of R(*t*)*^j^*, denoted R(*t*)*^5000^, and its 95% CI is R(*t*)*^250^–R(*t*)*^9750^.

To clarify associations among R(*t*) and the GTTC or other variables in addition to climate, mobility, and countermeasures, we used ordinary least-squares regression to regress the daily R(*t*) on daily dummy variables for the GTTC (G(*t*)); daily data of airport limousine bus users and visitors at the resort hotels (L(*t*)); as well as dummy variables for daily climate (H_1_(*t*) for temperature and H_2_(*t*) for humidity), mobility (M_i_(*t*) (i=1-6)), and the second state of emergency as follows:

R(*t*) = α + βG(*t*) + γL(*t*) + δ_1_H_1_(*t*) + δ_2_H_2_(*t*) + Ση_i_M_i_(*t*) + θP(*t*) + ε(*t*)

The start of the study period was June 20, 2020. Therefore, school closure and voluntary event cancellation and the first state of emergency had ceased; accordingly, we are unable to estimate their effects on R(*t*).

We anticipated the following influence of the explanatory variables: airport limousine bus users and visitors at the resort hotels or part of the GTTC would contribute to increased infectivity if the policy banning long-distance domestic travel was rational, and countermeasures such as the emergency state or school closure and voluntary event cancellation were presumed to decrease infectivity. We adopted 5% as the significance level and performed all statistical analyses using Stata SE 17.0 software (Stata Corp).

### Ethical Considerations

Information about the number of patients used for this study was collected under the Law of Infection Control, Japan, published by the Kagoshima Prefectural Office [34]. Iwasaki Industrial Corp Ltd provided the number of airport limousine bus user data from their business records. Both data sets only provided the number of persons, and thus did not include private or privacy information. There were therefore no ethical issues related to this study.

## Results

Figure 1 shows the numbers of newly confirmed cases of COVID-19, including asymptomatic cases, in Kagoshima from March 26, 2020, to February 28, 2021. The initial case was detected in Kagoshima on March 26. However, data were sporadic in the initial phase. From June 2020, cases were reported continuously.

**Figure 3:**
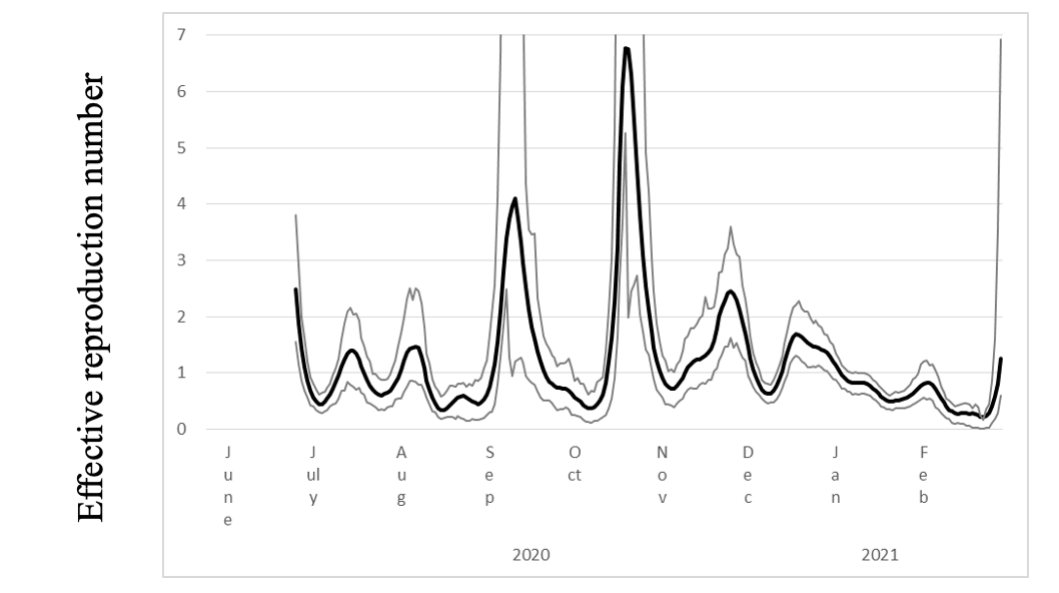
Effective reproduction number (black line) with 95% CIs (gray lines) of COVID-19 in Kagoshima prefecture, Japan, from June 20, 2020, to the end of February, 2021.

Figure 3 presents the estimated R(*t*) and 95% CI for the study period. Before June 2020, the R(*t*) was large and volatile because very few cases were reported. After June 2020, because new cases were reported almost daily, the R(*t*) became smaller and exhibited less volatility. The largest peak was in November 2020 while the GTTC was in effect.

**Figure 4:**
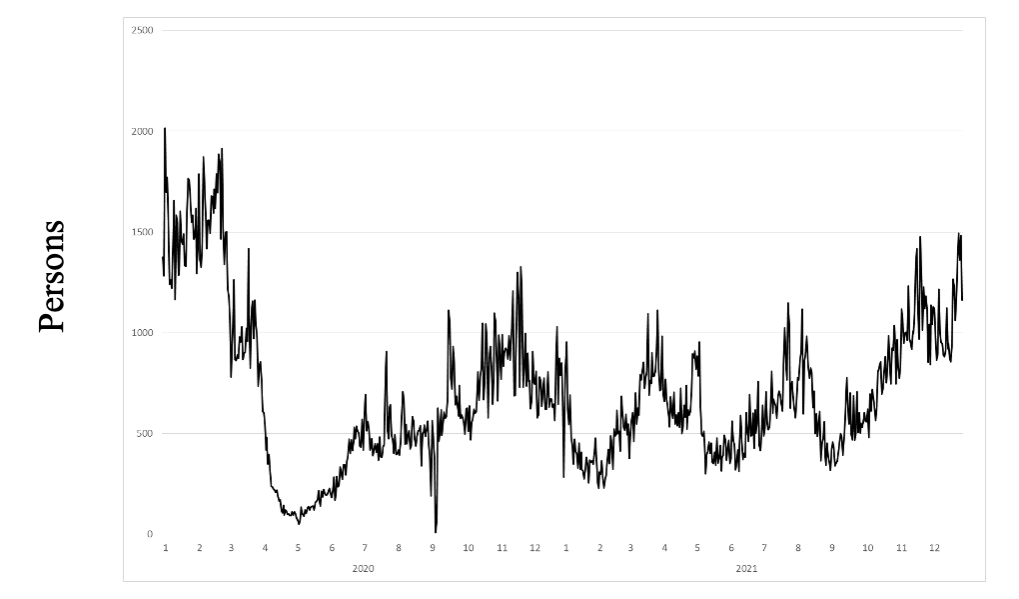
Number of airport limousine bus users at Kagoshima airport from the beginning of 2020 to the end of 2021.

Figure 4 portrays the number of the airport limousine bus users during the study period. The main peak of airport limousine bus users occurred before the outbreak emergence. During April and May of 2020, when the first state of emergency was declared, this number decreased considerably, reaching 0 in September 2020 when the airport was closed because a typhoon struck the area.

Table 1 presents the estimation results (adjusted *R*^2^=0.2772; n=273 observations) from the regression model. Climate conditions, including temperature and humidity, did not show any significant associations with R(*t*). In addition, there was no association of specific places with R(*t*) over the shorter period, except for restaurants and grocery stores. One can infer that going to a restaurant increased infectivity, whereas going to a grocery store, perhaps as a reflection of “staying home,” reduced infectivity. This result might indicate that a “stay-at-home” policy, including a lockdown or voluntary ban against going out as practiced in Japan, was legitimate. However, staying at “home” itself and going to a “workplace” were not found to be significant factors influencing R(*t*), even though they had negative coefficients. The first and second states of emergency and the GTTC had negative and significant effects on R(*t*). In particular, the estimated coefficients of these variables were quite large. The second state of emergency, which was not applied to Kagoshima, also showed an association with reduced infectivity.

**Table 1:** Estimated effects of various factors on infectivity (effective SARS-CoV-2 reproductive number) obtained using data from June 20, 2020, to the end of February 2021 In Kagoshima prefecture, Japan.

Explanatory variable		Estimated coefficient	*P* value
Airport limousine bus users		–0.003	.02
Temperature		0.013	.76
Humidity		0.003	.89
**Places**			
	Restaurant, shopping mall, or amusement center	0.094	.03
	Grocery store or pharmacy	–0.095	.03
	Park	–0.038	.08
	Transition	0.028	.52
	Workplace	–0.035	.44
	Home	–0.303	.09
Second state of emergency		–3.774	<.001
GTTC^a^		–3.040	<.001
Constant		7.646	<.001
*F* value (*df*=11, 261)^b^		10.48	<.001

^a^GTTC: “Go To Travel” campaign.

^b^The *F* test was used to evaluate the null hypothesis that all coefficients except for constant terms are 0.

## Discussion

### Principal Findings

Estimation results for the GTTC and the number of airport limousine bus users indicate that the promotion of long-distance domestic travel might decrease SARS-CoV-2 infectiousness. This finding thus appears to be inconsistent with a legitimate policy banning long-distance domestic travel, including cessation of the GTTC. Our findings suggest that during sightseeing or long-distance domestic travel, tourists/visitors and hosts might be much more conscientious about infection control and might therefore be less likely to infect others than when they are in their hometown. These phenomena were also indicated through a psychographical study and market research [37,38]. In other words, people in their hometown might exhibit less conscientious behaviors and might therefore be more likely to become infected. Therefore, discouraging long-distance domestic travel might actually engender worse infection rates overall within a local area.

This finding is consistent with earlier studies [35,39]. One study showed that the GTTC reduced infectiousness, whereas the other study found that events with an audience might not raise infectiousness compared to events without an audience.

Another study [40] using two types of patient data (onset date and the date of testing positive) found much higher travel-associated COVID-19 incidence during the period of July 22-26, when the GTTC was initiated, than during either an earlier period of June 22 to July 21, July 15-19, or June 22 to July 21 in terms of the incidence rate ratio (IRR). The same study also compared the period of August 8-31.

Some notable points can be identified from this previous study [40]. First, the proportion of people with a travel history during the GTTC period was comparable to that during the two prior periods. In particular, the proportion of people with a travel history among patients with COVID-19 who had an available onset date was smaller for the GTTC period than during the prior period of July 15-19. However, the authors found a significantly higher COVID-19 incidence at the beginning of the GTTC. These findings might merely reflect the fact that the total number of patents in the GTTC period was higher than that during the prior period. In other words, they did not control for the underlying outbreak situation and therefore found an incorrect association. Use of the IRR would be valid if the underlying outbreak situation other than the examining point was the same in the two considered periods. Therefore, application of the IRR might be inappropriate for addressing this issue. At the very least, controlling for the potential differences in the outbreak situation is considered to be necessary. The underlying outbreak situation, unrelated to the GTTC, was reflected in the number of patients without a travel history or any sightseeing. One potential approach to control for the underlying outbreak situation is to consider the share of patients with a travel history or sightseeing. However, that share did not increase markedly during the initial stage of the GTTC. This lack of a marked increase indicates that the authors’ results and conclusions are misleading.

Second, Anzai and Nishiura [40] referred to the period of August 8-31, 2020, when the GTTC was still in effect. The proportion of patients with a travel history was much smaller than that during the period of July 22-26 when the GTTC started or in the prior period. Although the authors did not compare the COVID-19 incidence in August with that of either the prior period or July 22-26 when the GTTC started, the rate of incidence in August 2020 was likely lower than that in other periods. In fact, some patients with active COVID-19 infections traveling under the GTTC might have been included in the study period, August 2020, as described above. Their inclusion might be inconsistent with the authors’ conclusion.

Third, we observed the peak of newly infected persons on July 23, 2020, which was the start date of the GTTC, for the entirety of Japan. Therefore, we infer that the GTTC might have reduced infectiousness. We also considered the potential effect of climate conditions on the variation in infectivity. At around the end of July, the rainy season in Japan ends and summer begins, accompanied by high temperatures. Therefore, the GTTC might have been insufficient to increase the number of COVID-19 patients and cancel out the benefits from the improved climate conditions. Taken together, these points suggest that the GTTC might not have been the main factor determining the course of the COVID-19 outbreak.

Moreover, if the GTTC did have a strong effect on the outbreak dynamics, then there would be an increase in the number of patients with no travel history. For example, one can consider a patient traveling under the GTTC on July 22 and 23, with disease onset occurring on July 24. Although this patient had a travel history in the GTTC period, they would not be included in a group of patients with a travel history whose onset date corresponds to the initial GTTC period of July 27-31. Nevertheless, presymptomatic patients are well known to become infectious during the symptomatic period [36]. In the above scenario, this patient might infect staff members of hotels or other individuals encountered in the visiting areas. However, if their onset dates were July 27 and 28, they would be included in the group of patients without a travel history in the GTTC start period of July 27-31. Therefore, the GTTC certainly increased the number of patients without a travel history but did not increase the number of patients with a travel history in this case. Consequently, when considering the effects of the GTTC, it is important to account for the total number of patients with COVID-19, irrespective of their travel history.

Finally, it is noteworthy that this study could have been performed in the middle or end of March 2021, if we had analyzed those data at that time. We found the same results as those found from this study. In fact, this analysis was performed in 2022, although similar research without the valuable data used for this study was posted to the medRxiv preprint server on January 4, 2021, and we obtained the same results for the GTTC [35]. In general, an ex ante policy evaluation is necessary, although it was very difficult to estimate its effects precisely. By contrast, an ex post evaluation performed as soon as possible could have been possible if such preparation had been arranged before policy activation. If such preparation had been done, then the policy banning long-distance domestic travel with no legitimate rationale could have been prevented in 2021 and thereafter.

Another study in Japan [41] showed that the GTTC was responsible for the introduction of an emerging sublineage of SARS-CoV-2 in October 2020 to Hokkaido, Japan’s second largest island. The ratio of the number of travelers at Hokkaido to that on the same month in the prior year was the largest in October 2020. However, this might not necessarily imply that the outbreak was accelerated by the GTTC or the number of travelers.

For this study, because daily airport user data were not available, we used the number of daily airport limousine bus users as a proxy of daily airport users, including those who did not use limousine buses. However, monthly airport user data have since been published [42]. Therefore, we further evaluated the representativeness of airport limousine bus users for airport users on a monthly basis. The correlation coefficient between monthly airport limousine bus users and airport users during 2020 and 2021 was 0.9881 (*P*<.001). Therefore, we can infer that airport limousine bus users constitute a good proxy of overall airport users. Moreover, even though bullet-train or bus services were available as a means of transportation to Kagoshima from neighboring or nearby prefectures, airlines are the only means of transportation to Kagoshima from areas with large populations in Japan, such as Osaka and Tokyo. Therefore, we can infer that airport limousine bus users are a good proxy of long-distance domestic travel volumes for Kagoshima.

This study excluded some variables suggested by earlier studies, such as vaccination, contact tracing, or mass gathering events, which potentially affect infectivity. Vaccination for COVID-19 started in March 2021 in Japan for health care workers. Therefore, there was no vaccination performed during our study period [43-45].

Moreover, contact tracing had been performed with the same intensity during the study period in Japan and was continued until the Omicron variant strain emerged. The public health center could not be traced with the same intensity. Contact tracing might be effective, at least in the very early stage, when the number of cases was limited to a few patients per public health center [46]. However, even in the very early stage of the pandemic in Japan, 80% of the infection sources were unknown. Therefore, contact tracing should not be expected to be effective in most cases in Japan [47]. Nevertheless, we were not able to estimate the effects or intensity of contact tracing for this study because it did not vary during the study period.

Mass gathering events such as the Olympic and Paralympic Games were also excluded from our analyses because of the study period. Even though international visitors seeking to see the games were refused because many players and officers were already crowded in a small area, an outbreak in the players’ village had been expected [48].

### Limitations

This study has some limitations. First, this study specifically assessed data from Kagoshima. Therefore, it remains unclear whether the same results would hold for other regions or for the entirety of Japan.

Second, we particularly examined the ancestral strain of SARS-CoV-2, which might be less infective than the Alpha variant strain [28-31] and the subsequent dominant Delta and Omicron variant strains [29,30,49,50]. Thus, the effects of a policy banning long-distance domestic travel might have been different under a scenario of the dominance of these variant strains.

Third, if complete daily information about long-distance domestic travel to Kagoshima prefecture were available, obviating the use of data particularly addressing only some travel, then the implications might differ from those obtained with this analysis. We consider that our data do reflect complete and precise travel information, although it is not possible to prove this at present.

Fourth, regression analyses such as that used for this study cannot demonstrate causality. Although we interpreted the number of airport limousine bus users as showing decreased infectivity, lower infectivity pushed up the number of airport limousine bus users. Therefore, the results need to be interpreted cautiously.

### Conclusion

We demonstrated that the GTTC or the increase of tourists and long-distance domestic travel visitors might not contribute to increasing COVID-19 infectiousness. Therefore, the policy banning long-distance domestic travel, including cessation of the GTTC, was neither fair nor rationally justified. Even though this analysis was performed much later than the study period of focus, the same results would be obtained considering the periods of April or May 2021 if we had performed this study at that time. The findings might have been helpful at that time for more rational decision-making when the government was considering whether to restart the GTTC. If so, then evidence-based policy might be suggested and operated. This perspective is in line with that of an earlier study [51].

## Abbreviations

GTTC: “Go To Travel” campaign
IRR: incidence rate ratio
R(*t*): effective reproduction number

## Data Availability

The data of airport limousine buses are not publicly available because they are sales data of the private company. However, the data are available from the corresponding author on reasonable request. Other data used for this study are available from the corresponding author.
